# An RBPJ-*Drosophila* Model Reveals Dependence of RBPJ Protein Stability on the Formation of Transcription–Regulator Complexes

**DOI:** 10.3390/cells8101252

**Published:** 2019-10-14

**Authors:** Bernd M. Gahr, Franziska Brändle, Mirjam Zimmermann, Anja C. Nagel

**Affiliations:** 1 Institute of Genetics (240), University of Hohenheim, Garbenstr. 30, 70599 Stuttgart, Germany; Bernd.Gahr@uniklinik-ulm.de (B.M.G.); Franziska.braendle@web.de (F.B.); zimmermann.mirjam@uni-hohenheim.de (M.Z.); 2 Present address: Molecular Cardiology, Department of Internal Medicine II, University of Ulm, Albert-Einstein-Allee 23, 89081 Ulm, Germany

**Keywords:** Notch signaling pathway, functional conservation, regulation, RBPJ, Su(H), *Drosophila*, model system

## Abstract

Notch signaling activity governs widespread cellular differentiation in higher animals, including humans, and is involved in several congenital diseases and different forms of cancer. Notch signals are mediated by the transcriptional regulator RBPJ in a complex with activated Notch (NICD). Analysis of Notch pathway regulation in humans is hampered by a partial redundancy of the four Notch receptor copies, yet RBPJ is solitary, allowing its study in model systems. In *Drosophila melanogaster*, the RBPJ orthologue is encoded by *Suppressor of Hairless* [*Su(H)*]. Using genome engineering, we replaced *Su(H)* by murine *RBPJ* in order to study its function in the fly. In fact, RBPJ largely substitutes for Su(H)’s function, yet subtle phenotypes reflect increased Notch signaling activity. Accordingly, the binding of RBPJ to Hairless (H) protein, the general Notch antagonist in *Drosophila*, was considerably reduced compared to that of Su(H). An H-binding defective *RBPJ^LLL^* mutant matched the respective *Su(H)^LLL^* allele: homozygotes were lethal due to extensive Notch hyperactivity. Moreover, RBPJ^LLL^ protein accumulated at lower levels than wild type RBPJ, except in the presence of NICD. Apparently, RBPJ protein stability depends on protein complex formation with either H or NICD, similar to Su(H), demonstrating that the murine homologue underlies the same regulatory mechanisms as Su(H) in *Drosophila*. These results underscore the importance of regulating the availability of RBPJ protein to correctly mediate Notch signaling activity in the fly.

## 1. Introduction

A small number of highly conserved signaling cascades governs the spatiotemporal development of multicellular organisms, including humans. The Notch signaling pathway is one of those, being involved in a multitude of cell fate decisions by mediating direct cell–cell interactions. Therefore, it comes as little surprise that various diseases, as well as different cancers, are associated with a dysregulation of Notch signal transduction (reviewed in [1,2]). Hence, expanding our knowledge of Notch regulation helps to further our understanding regarding the pathology and therapeutic control of Notch dependent diseases. Notch signal transduction is initialized by the binding of ligands to the receptor Notch, resulting in the cleavage and release of the intracellular Notch domain NICD from the membrane. No further intermediate steps are needed to transmit the Notch signal to the nucleus, as NICD itself is involved in the transcriptional response of Notch target genes. This response is realized by the association of NICD with the nuclear effector CSL and the co-activator Mastermind (Mam): together, they assemble a ternary Notch activator complex that governs the transcription of Notch target genes (reviewed in [1,3,4]). In addition, associated factors actively shape the chromatin landscape to a more open conformation typified by active chromatin marks, like histone H3 Lys27 acetylation or histone H3 Lys4 monomethylation. These involve histone acetyltransferases like CBP/p300 and histone methylases like KMTD2 (*trithorax-related, trr* in *Drosophila*) that are recruited by the activator complex in response to Notch activation [2,5,6,7]. In the absence of Notch signaling activity, CSL assembles a repressor complex that recruits further co-repressors, including histone deacetylases and H3K4 demethylases, for example, Kdm5 (*little imaginal discs, lid* in *Drosophila*) and Lsd1 (*suppressor of variegation 3-3, Su(var)3-3* in *Drosophila*) [8,9,10,11] (reviewed in: [2,5,12]). 

**Figure 1:**
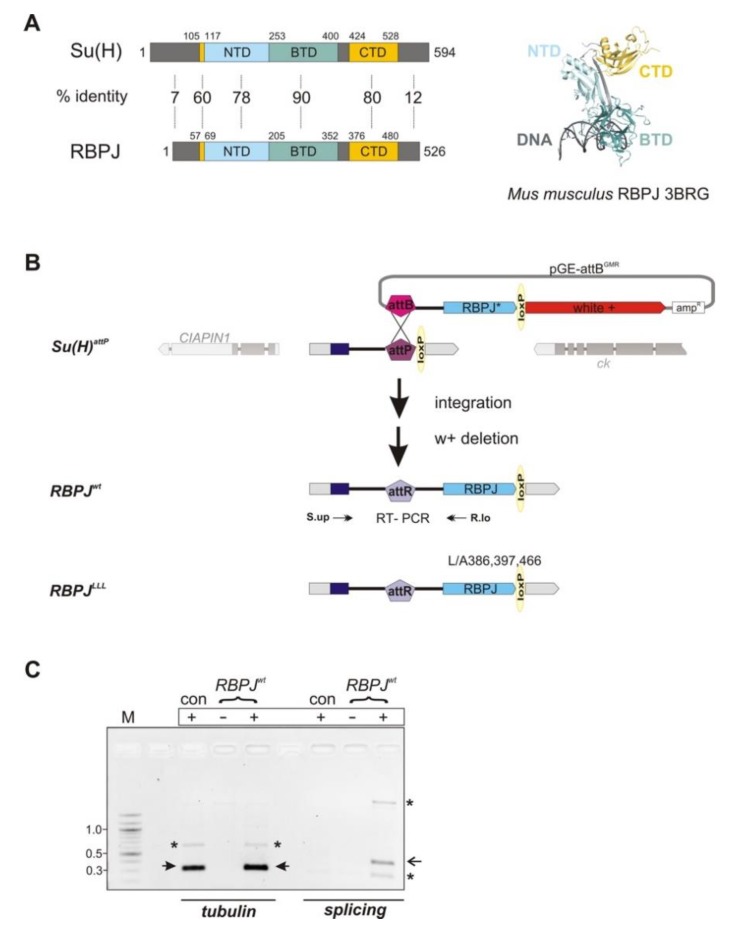
Genome engineering at the *Su(H)* locus to integrate murine *RBPJ*. (**A**) Comparison of *Drosophila* Su(H) protein with RBPJ protein from *Mus musculus*. N-terminal domain (NTD, light blue); ß-trefoil domain (BTD, green); C-terminal domain (CTD, yellow) and the N-terminally located alpha1-helix, which makes contact to CTD (yellow), are well conserved with identity between 60% and 90%. The flanking parts of the proteins, however, show little conservation. Numbers above the proteins depict codons, framing the respective domains. Structure of RBPJ bound to DNA (PDB ID: 3BRG) [38]. DNA is colored in gray; domains in RBPJ are colored as above. (**B**) Flow chart of strategy used to exchange *Su(H)* with murine *RBPJ* by genome engineering. The founder line *Su(H)^attP^* was used to integrate *RBPJ^wt^* and *RBPJ^LLL^* cloned in pGE-attB^GMR^ via *PhiC31-*integrase mediated recombination at the attP landing site. Subsequently, vector sequences and the *white^+^* marker, flanked by loxP sites, were excised with the help of the *Cre*-recombinase to yield the final fly strains *RBPJ^wt^* and *RBPJ^LLL^*. (**C**) Splicing of *RBPJ^wt^* mRNA occurred as expected in the *RBPJ^wt^* strain, leading to a PCR product of about 410 bp (open arrow). RT-PCR was performed on cDNA from the given strains, using *y^1^ w^67c23^* flies as controls; (+) with reverse transcriptase and (–) no-RT control. Primer pairs *S.up* and *R.lo* are depicted schematically in (B). Tubulin primers served as controls for intact mRNA (arrows). As size standard (M), a 100 bp ladder was used. ^*^ Label unspecific bands.

Analysis of the Notch pathway regulation in humans is hampered by partial redundancy: there are four copies of the Notch receptor, and two to three copies of the two different Notch ligands (reviewed in [3]). The signal transducer CSL is solitary; however, defects in CSL are expected to be fatal since all Notch signaling activity is affected. In fact, CSL mutant mice are embryonic lethal, and conditional mutants are unfortunately lacking [13], essentially preventing detailed analysis of CSL regulation in mammals. The CSL transcription factor is highly conserved in evolution, allowing analyses in simpler model systems. Structural information available from mice, worms, and flies shows remarkable similarities between the CSL homologues [14,15,16]. CSL proteins are subdivided into three major domains: the N-terminal domain (NTD) and the C-terminal domain (CTD) are separated by the ß-trefoil domain (BTD), with an N-terminal alpha-helix reaching into the CTD (Figure 1A and Figure S1). DNA binding is mediated by the NTD and BTD domains, whereas NICD interacts with the BTD and CTD, and Mam with the NTD and BTD to assemble a ternary activator complex (reviewed in [4]). Originally identified as *‘Recombination signal binding protein for immunoglobulin kappa J region*’ (RBPJ) in mammals, CSL is represented by *Suppressor of Hairless* [Su(H)] in *Drosophila* [17,18]. RBPJ, and Su(H) show the highest similarities in the BTD and CTD (BTD, 90%/94% and CTD, 80.5%/90.5% identity/similarity, respectively), whereas it is rather low outside of these domains (around 20% similarity only) (Figure 1A and Figure S1). This high conservation among CSL proteins raised our expectations as to the functionally of replacing *Drosophila* Su(H) with mouse RBPJ, in order to generate a ’mammalian’ fly model for future studies on RBPJ regulation.

Similar to its orthologues in other organisms, Su(H) is not only key for Notch mediated target gene activation but also for its silencing; thus, it is often described as the molecular switch during Notch signal transduction. In the absence of Notch activity, RBPJ/Su(H) mediates the silencing of Notch target genes by recruiting various co-repressors. However, the modus operandi seems to differ between mammals and *Drosophila* [19]. Whereas several co-repressors exist in mammals, which compete with NICD for binding directly to the BTD domain in RBPJ, the best characterized co-repressor in *Drosophila*, Hairless (H), interacts rather with the CTD of Su(H) (reviewed in [4,5,12,19]). Although not direct competitors, H impedes the binding of NICD through a conformational change in the structure of Su(H) [20,21]. The interface of binding between Su(H) and H was mapped to three leucines (L434/L445/L514) located in the hydrophobic core of the CTD in Su(H), which, when mutated, destroy H/Su(H) repressor complex formation but still allow Notch activator complex formation [21]. Intriguingly, although the mechanism of Notch silencing differs, murine RBPJ was shown to physically interact with H in vitro [20,22], suggesting that it may be able to form repressor complexes like Su(H). In agreement, the constitutive overexpression of murine RBPJ allows for the survival of *Su(H)* mutant animals, suggesting a largely functional replacement of *Su(H)* by RBPJ in the fly [23]. Moreover, the regulation of chromatin accessibility in response to Notch activity or inactivity is highly conserved between flies and mammals, mediated by a conserved set of histone-modifying enzyme complexes and histone chaperones, as well as cooperating transcription factors (reviewed in [2,5,12]). 

In this study, we replaced the *Su(H)* gene with the murine *RBPJ* homologue at its native locus, by genome engineering, to address the functional substitution in the natural cellular environment and regulation in the fly. The resultant *RBPJ^wt^* flies are homozygous viable; however, they show signs of a moderate increase of Notch activity in different Notch dependent processes. This conforms to the reduced repressor ability of RBPJ in the fly, explained by its mitigated binding affinity to H. In contrast, an H-binding deficient variant *RBPJ^LLL^* is lethal, demonstrating the necessity for H interaction and repressor complex assembly during fly development. Moreover, *RBPJ^LLL^* mutants are not only characterized by a hyper-activated Notch signaling readout but also, similar to the *Su(H)^LLL^* mutant, by a decreased abundance of RBPJ^LLL^ protein. Altogether, stability of RBPJ protein in the fly appears similar to Su(H) protein, emphasizing the role for transcriptional complexes in protein stabilization. Hence, the regulation of CSL protein availability may be a more general principle for balancing Notch signaling activity during development.

## 2. Materials and Methods

### 2.1. Generation of Mouse RBPJ Constructs and Establishment of RBPJ^wt^ and RBPJ^LLL^ Mutant Flies

A pGEX-6-p1-mouse *RBPJ* clone containing the 1645 bp full-length mouse *RBPJ* cDNA fused to C-terminal 6xHis tag was kindly provided by R. Kovall, University of Cincinnati, USA. This clone was used as a template for PCR amplification using Q5 High-Fidelity Polymerase (New England Biolabs, Frankfurt, Germany) and the following primer pair: UP: 5’ **GAA TTC CAG** GTG GCA CAG AAG TCT TAC GGA AAT G 3’; LP: 5’ GCG GCC GCT CGA G***TT A****GT GGT G* 3’. The primers contained the *Su(H)* intron sequences and the splice acceptor (bold in UP) N-terminal of valine 81 of mouse *RBPJ*, as well as 2x His tags (italics in LP), followed by the stop codon (bold and italics in LP). To allow shuttling, oligos contained restriction sites *Eco*RI in the UP and *Xho*I in the LP (underlined). The *RBPJ* PCR product was first cloned into the StrataClone^TM^ PCR cloning vector pSC-B (Stratagene, La Jolla, Ca, USA) and afterward shuttled into *Eco*RI/*Xho*I opened pBT∆NEP *gSu(H)* clone, maintaining the first exon and intron of *Su(H)* [24]. Finally, an 1882 bp *Bam*HI/*Xho*I fragment containing the first intron of *Su(H)* and the entire mouse *RBPJ* cDNA was shuttled into *Bgl*II/*Xho*I digested pGE-attB^GMR^ [25], to be inserted into the attP site of the founder line, as outlined before [24,26]. To this end, embryos at the pre-cellular blastoderm stage, derived from a cross of males w^*^; *Su(H)^attP^*/CyO-GFP [24] and females *y^1^* M{vas-int.Dm}ZH-2A w^*^ (BL40161) expressing the PhiC31 integrase, were injected, and offspring were selected based on red eyes, to be further verified by diagnostic PCR [24,25,26,27,28]. Subsequently, the *white^+^* marker gene and vector sequences were deleted with Cre-recombinase by a cross with *y^1^ w^67c23^* Sco/CyO,P{Crew}DH1 (BL1092). White-eyed offspring without recombinase were selected [25,28] and balanced with CyO-GFP. Substitution mutations (leucine 386, 397, and 466 by alanine) were introduced into the *RBPJ* cDNA by PCR-mutagenesis, using the Site-Directed Mutagenesis Kit from New England Biolabs (Frankfurt, Germany) and sequence specific mutagenesis primer pairs. Subsequent generation of *RBPJ^LLL^* mutant flies followed the steps described above for the wild type form. DNA constructs were sequence verified (Macrogen Europe, Amsterdam, Netherlands). Genotypes of the resultant flies were confirmed by PCR, diagnostic restriction digests, and sequence analysis. 

### 2.2. Fly Work and Documentation of Adult Phenotypes

Stocks were kept on standard fly food at 18 °C. The survival rate was assayed at 25 °C, using 15–20 virgins per cross to avoid overcrowding of the cultures. To increase the number of homozygous *RBPJ^wt^* and *RBPJ^LLL^* larvae, flies were cultured at 25 °C on enriched food (10 g agar, 0.5 g CaCl_2_, 60 g glucose, 20 g yeast extract, 0.5 g MgSO_4_, 20 g peptone, 30 g sucrose, 80 g dry yeast, and 6 ml propionic acid per liter). The *RBPJ* mutants were balanced over CyO-GFP to allow for the selection of homozygous mutants devoid of GFP, using a Leica UV-dissecting microscope MZ FL III with GFP filter set. As controls, we used *y^1^w^67c23^* (BL6599), *Su(H)^attP^*, *Su(H)^gwt^*, and *Su(H)^LLL^* [24]. Scanning electron micrograph pictures of adult flies were documented with a tabletop NeoScope (JCM-5000; Nikon, Tokyo, Japan). Adult wings from females were dehydrated in ethanol and mounted in Euparal (Roth, Karlsruhe, Germany), to be documented with an ES120 camera, (Optronics, Goleta CA, USA) connected with a Zeiss Axiophot microscope (Carl Zeiss AG, Jena, Germany) using Pixera Viewfinder software, version 2.0. To induce Flp/FRT-based mosaics, FRT40A was recombined with the *RBPJ* alleles and crossed with y^1^w* hs-flp; *P{w^+mC^ = Ubi-GFP.D}33 P{w^+mC^ = Ubi-GFP.D}38 P{ry^+t7.2^ neo-FRT}* 40A/CyO (BL5189). Early second larval offspring were heat-shocked for one hour at 37 °C and prepared for antibody staining at the third instar larval stage. Fly strains used in this work were as follows: *y^1^* M{vas-int.Dm}ZH-2A w^*^ (BL40161), *y^1^ w^67c23^* Sco/CyO,P{Crew}DH1 (BL1092), *y^1^w^67c23^* (BL6599), w^*^; *Su(H)^attP^*/CyO-GFP [24], w^*^; *Su(H)^gwt^* [24], w^*^; *Su(H)^LLL^*/CyO-GFP [24], w^*^; *RBPJ^wt^*/CyO-GFP (this work), w^*^; *RBPJ^LLL^*/CyO-GFP (this work), w^*^; FRT40A *Su(H)^attP^*/CyO-GFP [24], w^*^; FRT40A *Su(H)^gwt^*/CyO-GFP [24], w^*^; FRT40A *Su(H)^LLL^*/CyO-GFP [24], *w^1118^*; P{white-un1}30C P{neoFRT}40A (BL1646), w^*^; FRT40A *RBPJ^wt^*/CyO-GFP (this work), w^*^; FRT40A *RBPJ^LLL^*/CyO-GFP (this work), y^1^ w* hs-flp; *P{Ubi-GFP.D}33 P{Ubi-GFP.D}38 P{ neo-FRT}* 40A (BL5189). 

### 2.3. Immunochemistry

Third instar larval wing discs were dissected and stained according to standard protocols. Briefly, discs attached to the mouth hook were prepared and fixed for 20 minutes in 4% paraformaldehyde, under gentle shaking. After several washes in PBX (PBS with 0.3% Triton X-100) and pre-incubation with 4% normal donkey serum, the discs were incubated with primary antibodies overnight at 8 °C. Primary antibodies used were mouse anti-Wg 4D4 (1:25), mouse anti-Pebbled (Hnt) 1G9 (1:10), developed by S.M. Cohen, and H. Lipshitz, respectively, and obtained from DSHB, the Developmental Studies Hybridoma Bank, developed under the auspices of the NICHD and maintained by the University of Iowa, Department of Biology, Iowa City, IA 52242); rabbit anti-GFP (1:50; Santa Cruz Biotech, Dallas, TX, USA), rabbit anti-RBPSu(H) (1:500; #5442 Cell Signaling Technology, Frankfurt, Germany); and rat anti-Deadpan (Dpn) (1:100; ab19573 Abcam, Cambridge, UK). After several washes with PBX, discs were pre-incubated with 4% normal donkey serum before adding secondary donkey antibodies coupled with FITC or Cy3 (Jackson Immuno-Research, obtained from Dianova, Hamburg, Germany). Incubation was either overnight at 8 °C or 2–3 hours at RT, followed by several washes in PBX. Dissected discs were mounted in Vectashield (Vector labs, Eching, Germany) and documented with a Zeiss Axioskop coupled with a BioRad MRC1024 confocal microscope, using LaserSharp 2000 software (Carl Zeiss, Jena, Germany). Pictures were assembled using Photo Paint, Corel Draw, and ImageJ software.

For the quantification of larval RBPJ protein expression, 10 homozygous third instar larvae were homogenized in 100 µl binding buffer (20 mM HEPES pH 7.6, 150 mM KCl, 2.5 mM MgCl_2_, 10% glycerol, 0.05% NP-40, 1 mM DTT, ROCHE complete ULTRA protease inhibitor mini tablet), and protein amounts were normalized by larval weight and Bradford assays. Larval extracts were subjected to Western blotting, and rabbit anti-RBPSu(H) (1:500; #5442 Cell Signaling Technology, Frankfurt, Germany) and mouse anti-beta-tubulin A7 (1:3000; developed by M. Klymkowsky; obtained from DSHB, Iowa, USA) were used for detection. Goat secondary anti-rabbit or anti-mouse antibodies coupled with alkaline phosphatase (Jackson Immuno-Research, obtained from Dianova, Hamburg, Germany) were used for detection. The blots were cut to detect in parallel RBPJ and beta-tubulin proteins separately from the same blot. For the quantification of signals, (n = 4) blots were evaluated with the *ImageJ* gel analysis program. Beta-tubulin was used as the internal standard, and RBPJ^LLL^ protein levels were compared with RBPJ^wt^ levels. Significance was tested using an unpaired Student’s *t* test. *** *p* < 0.001. 

### 2.4. RT-PCR and Quantitative RT-qPCR

Correct splicing of RBPJ was confirmed by RT-PCR, which was performed as outlined before [24,29], using the primer pair *S.up* 5’ CCG GCC ACA CAT CGA GGA GAA G 3’ and *R.lo* 5’ CCG CTT GCT GAG GAA CAC ACC A 3’ and Tubulin56D primers the as controls. Quantitative RT-PCR was performed on four biological and two technical replicates of each genotype, using isolated wing imaginal discs from 20 homozygous wandering third instar larvae. Poly(A)^+^ RNA was prepared with the *Dynabeads^TM^ mRNA DIRECT^TM^ Micro Purification Kit* (Invitrogen, Thermo Fisher Scientific, Waltham, USA). cDNA synthesis and real-time qPCR were performed as described before [30]. As internal reference genes, *cyp33* and *Tbp* were used. The following primer pair sequences (given in parentheses) are listed at DRSC FlyPrimer bank [31]: *cyp33* (PP14577), *dpn* (PP17352), *E(spl)mß* (PP8427), and *Tbp* (PP1556). Other primers used were *peb* UP, 5’ GAG CGG CCA TTC CAG TGT GA 3’ and *peb* LP, 5’ TTG TTG TTG GCG CTG GTC GG 3’. Relative quantification of the data was performed with micPCR^®^ software version 2.8, based on REST [32], taking target efficiency into account. Expression values p < 0.05 are considered to be statistically significant.

### 2.5. Yeast Protein–Protein Interaction Assays

Yeast protein–protein interaction assays were based on the Golemis–Brent hybrid system, using EGY48 yeast cells (kindly provided by E. Golemis, Harvard Medical School) [33]. Details on the handling, strains, vectors, and media are outlined in [34,35]. Binary protein–protein interaction assays were performed with constructs cloned in pEG202 vector, allowing fusion with the LexA DNA-binding domain, and, in pJG4-5 vector, providing the B42-AD. pSH18-34 served as the reporter, expressing lacZ upon productive interaction [33,34]. To investigate ternary activator complex formation, Notch intracellular domain was expressed, in addition to pESC-Leu vector (Stratagene), as described earlier [20,21]. Media-lacking leucine allowed a selection for pESC presence. All experiments were done in triplicate. Quantitative yeast two-hybrid studies were performed according to [36]. Interaction strength was measured in Miller Units calculating the ratio of substrate turnover to cell density (1000 x OD_420nm_/time (min) x volume (ml) x OD_600nm_). Six independent clones were measured from two independent experiments. pEG-HFL encodes full-length *Drosophila* Hairless [37], pEG-Mam, and pESC-RICN, the relevant domains of *Drosophila* Mastermind and Notch, respectively [21]. pEG-MamL and pESC-NICD from *Mus musculus* were cloned by using PCR-amplified products of the respective encoding domains. pEG-MamL contains codons alanine 12 to histidine 74, derived from Mastermind-like protein 1, subcloned as *Bgl*II/*Xho*I fragment in pEG202 opened with *Bam*HI/*Xho*I. pESC-NICD includes codons arginine 1751 to serine 2293, derived from Notch homolog protein 1 precursor, subcloned as *Spe*I/*Sac*I fragment in likewise opened pESC-Leu vector. The respective template DNA was kindly provided by F. Oswald (University of Ulm, Germany). Constructs were sequence verified (Macrogen Europe, Amsterdam, Netherlands). The pJG-constructs containing Su(H), Su(H)^LLL^, RBPJ^wt^, and RBPJ^LLL^ are all described in [21]. Primer sequences used for amplification (restriction sites used for cloning are highlighted):

NICD mouse UP: 5’ -GAT ***GAA TTC*** CGA CGA CGA CAT GGC CAG CTC TTG T-3’;
NICD mouse LP: 5’ -CGG ***GTC GAC*** TTA GCT TGC TGG TGC ACC CAC G -3’;
MamL UP: 5’ -GAT ***AGA TCT*** CCA TGG CAC TGC CGC GGC ACA-3’; and
MamL LP: 5’ -CTT ***CTC GAG*** TTA GGT GGC GAT GGA TCC CGG G-3’.

### 2.6. Statistical Analysis 

The statistical analysis was performed by Student’s *t* test or by ANOVA, using a two-tailed Dunnett’s or Tukey–Kramer approach for multiple comparisons. *** *p* < 0.001 highly significant; ** *p* < 0.01 very significant; * *p* < 0.05 significant; *p* > 0.05 not significant (ns). Box plots were assembled with the online tool BoxPlotR (http://shiny.-chemgrid.org/boxplotr/).

## 3. Results

### 3.1. Replacement of the Su(H) Locus with Murine RBPJ by Genome Engineering in Drosophila

With the aim to assess the biological activity of the murine orthologue *RBPJ* in the fly, the endogenous *Su(H)* locus was replaced by applying genome engineering, as outlined before [24,25]. To this end, we made use of the previously generated founder line *Su(H)^attP^*, allowing the introduction of constructs encoding *RBPJ* and a mutant variant thereof under the transcriptional regulation of the endogenous *Su(H)* gene. In *Su(H)^attP^*, most of the coding region is replaced by an attP landing site (Figure 1B) [24]. Due to the conceptual design, the first exon of Su(H) was retained, including the translation start and 128 amino acids that lie mostly within the non-conserved N-terminal part of the protein (Figure 1A,B and Figure S2). As a result of the integration event, an attR site was generated and most of the intron is duplicated; however, neither was touching the splice donor nor the splice acceptor sites (Figure 1B and Figure S2) [24]. Correct splicing was confirmed in the *RBPJ^wt^* flies by RT-PCR (Figure 1C).

Successful integration is expected to produce a fusion gene and protein, with the first exon and intron of *Su(H)*, followed by the coding region of *RBPJ*, starting with valine at position 81 of the mouse RBPJ (Figure S2). In addition, we generated a *RBPJ^LLL^* mutant version by substituting three conserved leucine residues (L386, L397, and L466) with alanine (Figure 1B and Figure S2). The respective residues in the Su(H) protein mediate the contact to the repressor Hairless (H) in *Drosophila* (Figure S1). When replaced by alanine, H binding is completely abolished [21,24]. Subsequent analyses on the newly established *RBPJ^wt^* or *RBPJ^LLL^* fly lines were performed after deletion of auxiliary vector sequences and the *white^+^* marker used for selection by Cre-mediated recombination (Figure 1B). The biological activity of the newly established *RBPJ^wt^* and *RBPJ^LLL^* lines was compared with the respective *Su(H)* alleles, i.e., *Su(H)^gwt^* carrying a likewise constructed *Su(H)* wild type gene copy in the attP landing site, as well as in the mutant *Su(H)^LLL^* version [24]. 

### 3.2. RBPJ^wt^ Flies Are Viable and Show Characteristics of a Gain in Notch Activity

**Figure 2:**
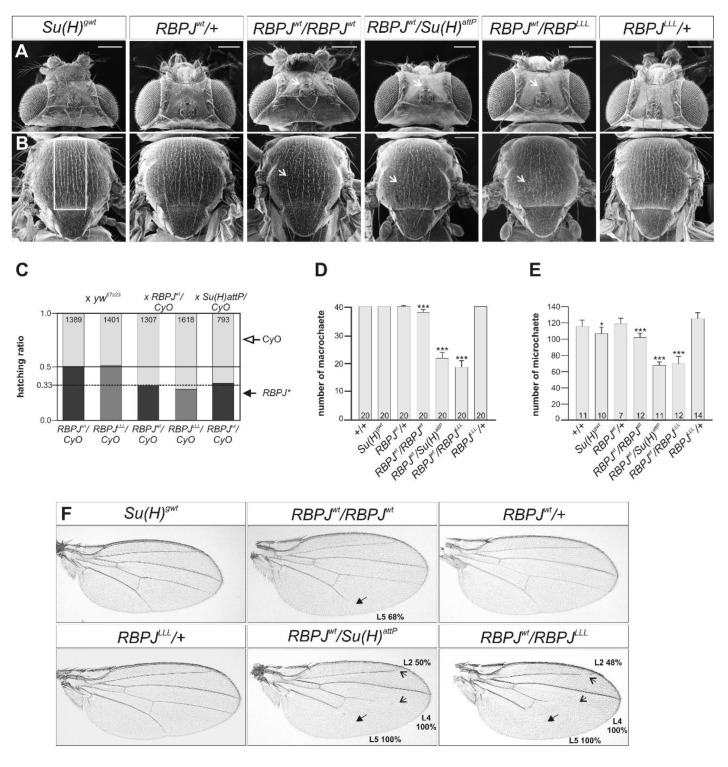
Adult phenotypes in *RBPJ* adults. (**A**,**B**) Scanning electron micrographs of fly heads (**A**) and thoraces (**B**) of the given genotype. In comparison to the control *Su(H)^gwt^* or the heterozygous *RBPJ^wt^* / +, the homozygous *RBPJ^wt^* flies have fewer macro- and microchaetae. This phenotype is enhanced in a *RBPJ^wt^/Su(H)^attP^* background or in the *RBPJ^wt^*/*RBPJ^LLL^* combination (arrows point to examples of missing bristles). *RBPJ^LLL^*/+ heterozygotes match the control. Scale bars: 200 µm. (**C**) Viability of heterozygous *RBPJ^wt^* and *RBPJ^LLL^* flies of the homozygotes and the *RBPJ^wt^/Su(H)^attP^* combination, respectively, was determined relative to their balanced siblings (hatching ratio). Bars depict the fraction of the expected offspring (*RBPJ^wt^* black, *RBPJ^LLL^* grey, and CyO light grey); numbers show total animals analyzed. The heterozygotes balanced over CyO were crossed to the flies with the genotypes given above. Note that *RBPJ^wt^* flies always hatch at the expected numbers, whereas *RBPJ^LLL^*/*RBPJ^wt^* heterozygotes are slightly underrepresented. (**D**) Average number of macrochaetae in adult females of the given genotype (n = 20). Note significantly reduced numbers in the homozygous *RBPJ^wt^* and *RBPJ^wt^/Su(H)^attP^* flies, as well as in the *RBPJ^wt^*/*RBPJ^LLL^* combination. (**E**) Average number of microchaetae determined from scanning electron micrographs; the evaluated sector is highlighted in the control in (**B**). Number of animals analyzed is given in each bar. Note significant reduction in *RBPJ^wt^*/*RBPJ^wt^* and *RBPJ^wt^/Su(H)^attP^* flies, as well as in the heterozygous *RBPJ^wt^*/*RBPJ^LLL^* animals. (**D**,**E**) Statistical analyses were performed with ANOVA Tukey–Kramer approach relative to wild type control (*** *p* < 0.001; * *p* < 0.05). (**F**) Typical examples of wings from female flies of the given genotype are depicted. Sixty-eight percent of the *RBPJ^wt^* homozygotes are characterized by a shortened L5 vein (arrow, n = 28), an effect which is enhanced in *RBPJ^wt^*/*Su(H)^attP^* flies, where additionally 100% of L4 and 50% of L2 veins are shortened (open arrows, n = 23). A likewise enhancement is seen in the *RBPJ^wt^*/*RBPJ^LLL^* combination (n = 29).

The *RBPJ^wt^* strain is homozygous viable, indicating that the *RBPJ* gene under the control of endogenous Su(H) regulatory elements is able to entirely replace *Su(H)* essential functions (Figure 2A,B). Accordingly, *RBPJ^wt^* homo- or heterozygotes appear with the same frequency as their siblings when crossed with *yw^67c23^* control or inter se (Figure 2C). Even in trans over null, i.e., *RBPJ^wt^*/*Su(H)^attp^*, flies emerged in the expected numbers of about 33% (Figure 2C). Closer inspection, however, uncovered some divergence from the wild type, indicating a slight increase in Notch activity. First, bristle numbers of both, micro- and macrochaetae were significantly reduced, notably in *RBPJ^wt^*/*Su(H)^attp^* flies (Figure 2A–E), which is typical of a failure in lateral inhibition during sensory organ precursors selection [39,40,41]. Second, most males were sterile due to abnormal genitalia orientation (Figure S3), a phenotype observed upon an increased Notch receptor activation and protein accumulation in genital discs [42]. Finally, wings of homozygous *RBPJ^wt^* flies displayed shortened longitudinal veins L5 with high penetrance (68%) (Figure 2F), which is characteristic of a gain of Notch activity [43,44]. Again, wing venation defects were enhanced in *RBPJ^wt^*/*Su(H)^attp^* animals (Figure 2F).

Apparently, *RBPJ^wt^* flies have a gentle increase in Notch activity rather than a lowered level. The increase, however, is too weak to impair the development of adult flies, but strong enough to produce subtle phenotypes. Some of the observed phenotypes resemble heterozygous *H* mutants (e.g., bristle and wing venation defects) [26,43,45,46]. Reduced numbers of microchaetae and rotation of male genitalia, however, were not yet linked to *H* mutations, but rather to a gain of Notch activity [42,43,44], suggesting a higher increase in Notch activity in the homozygous *RBPJ^wt^* compared to an *H* heterozygote. Overall, the data suggest that *RBPJ^wt^* has a defective regulation of Notch signaling activity, which may, for example, result from a gain in the activation of Notch targets or, alternatively, from impaired repression.

### 3.3. RBPJ^LLL^ Flies Are Lethal Due to a Failure of Repressor Complex Assembly

In contrast to *RBPJ^wt^*, *RBPJ^LLL^* mutants in homozygosis or over null (*RBPJ^LLL^*/*RBPJ^LLL^* and *RBPJ^wt^*/*Su(H)^attp^*) did not develop to adulthood but died at larval-to-pupal stages, as described for the *Su(H)^LLL^* mutant allele [24]. When heterozygous over a *Su(H)* wild type allele, *RBPJ^LLL^* animals emerged at the expected ratio, but with slightly reduced numbers when heterozygous over *RBPJ^wt^* (Figure 2C). Likewise, bristle numbers were significantly reduced in the *RBPJ^wt^* background but were normal in a *Su(H)* wild type background (Figure 2A–E). Moreover, whereas the heterozygote displayed normal wings, a combination with *RBPJ^wt^* revealed strong venation defects with fully penetrant shortened L5 and L4 veins and defective L2 in about half of the wings (Figure 2F). The phenotypes of *RBPJ^wt^*/*Su(H)^attp^* and *RBPJ^wt^*/*RBPJ^LLL^* animals were nearly indistinguishable, indicating that the *RBPJ^LLL^* allele did not contribute at all to RBPJ function with regard to adult fly development (Figure 2A–F).

The observed phenotypes support the idea that RBPJ protein can functionally replace Su(H) in fly development, albeit gaining Notch activity, perhaps by a failure to achieve full repression of Notch activity. In contrast, however, RBPJ^LLL^ cannot substitute for Su(H) function, indicating that assembly of RBPJ in H-mediated repression complexes is an absolute requirement for fly survival. Hence, RBPJ can only comply with the role of Su(H) upon binding to Hairless, just like its fly orthologue.

### 3.4. The Interaction between RBPJ and Hairless Is Impaired

Crystal-structure analysis, accompanied by in-depth interaction studies, provided a comprehensive view of the relevant residues in Su(H) required to build the Su(H)-H repressor complex [21,47]. Yeast two-hybrid studies mapped three leucine residues, L434, L445, and L514, within the Su(H) C-terminal domain that, when mutated to alanine, abrogated binding to H [21]. Interestingly, the mouse orthologue RBPJ was also shown to interact with H in a yeast two-hybrid approach, although no H homologue was identified so far in vertebrates [19,20]. Using yeast protein–interaction assays, we aimed to determine and quantify the ability of RBPJ to form activator or repressor complexes with the respective *Drosophila* components, since the phenotypic data had uncovered a gain of Notch activity in the *RBPJ^wt^* flies. We expected either an increase in the binding of RBPJ to intracellular Notch with or without Mam, or a decrease in the binding to the Notch antagonist Hairless, since either should result in stronger Notch target gene activation. 

**Figure 3:**
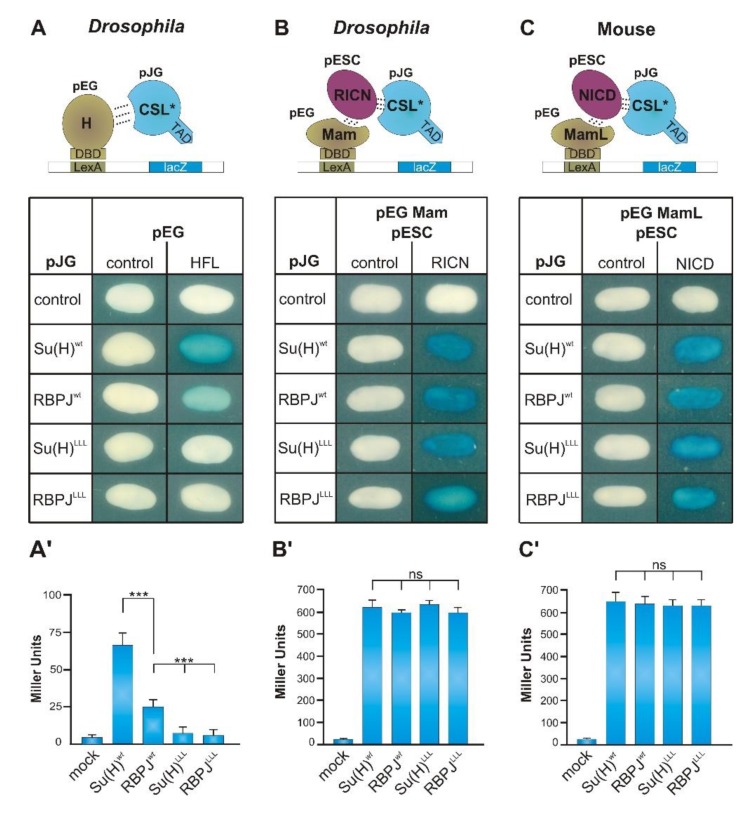
Assembly of repressor and activator complexes by RBPJ. (**A**) Yeast two-hybrid assay for the interaction of H with Su(H) and RBPJ variants, respectively. H fused to the lexA-DNA binding domain (DBD) provided in pEG vector; CSL variants fused to the trans-activator domain (TAD) in pJG vector. Interaction results in transcription of the lacZ reporter, as shown in the scheme. No binding is seen between the H-binding deficient Su(H)^LLL^ and RBPJ^LLL^ isoforms, whereas both Su(H)^wt^ and RBPJ^wt^ show binding, however the latter is much weaker than Su(H)^wt^. Interaction assays were done with the corresponding full-length proteins. (**B**) Yeast protein three-hybrid assay for formation of a ternary complex with the *D. melanogaster* components, co-activator Mam (aa 118–194) provided in pEG vector, RICN (intracellular Notch including RAM domain; aa 1762–2176) provided in pESC vector, and CSL variants provided in pJG vector. Ternary complex formation results in transcription of the lacZ reporter, as shown in the scheme. (**C**) Yeast protein three-hybrid assay as in (B) with *M. musculus* components, co-activator MamL (aa 12–74) provided in pEG vector, NICD (aa 1751–2293) provided in pESC vector, and CSL variants provided in pJG vector. (**A’**–**C’**) Quantification of the interactions shown in (**A**–**C**) is given in Miller Units. At least six different clones from two independent experiments were quantified and statistically analyzed with ANOVA and two-tailed Tukey–Kramer test relative to RBPJ. (*** *p* < 0.001; ns: not significant).

To this end, we assayed the protein–protein interaction of mouse RBPJ^wt^ or RBPJ^LLL^ with full-length Hairless in a two-hybrid assay (Figure 3A). Moreover, ternary activator complex formation was addressed in a modified protein three-hybrid assay, as outlined before [20,21,48,49] (Figure 3B,C): RBPJ^wt^ or RBPJ^LLL^ was co-expressed with the intracellular Notch domain (RICN) plus Mam from the fly, as well as with NICD plus MamL from the mouse (Figure 3B,C). We also included Su(H)^wt^ and Su(H)^LLL^ to allow a direct comparison of the two CSL orthologues from fly and mouse. We performed a qualitative analysis to demonstrate the interactions (Figure 3A–C) and, in addition, a quantitative analysis to measure the differences (Figure 3A’–C’). This analysis confirmed that the interaction strength between Hairless and mouse RBPJ was only about 40% of that between H and Su(H) (Figure 3A,A’). Moreover, we confirmed the complete lack of H-binding with either RBPJ^LLL^ or Su(H)^LLL^ protein, strongly indicating that an RBPJ-H repressor complex matches the structure of the Su(H)-H repressor complex, as predicted by the strong conservation between RBPJ and Su(H) (Figure 3A,A’, and Figure S1). At the same time, we could neither detect qualitative nor quantitative differences in activator complex assembly (Figure 3B–C’): all four CSL proteins, Su(H) or Su(H)^LLL^ and RBPJ^wt^ or RBPJ^LLL^, were indistinguishable in their ability to bind to NICD or to assemble the trimeric activator complex. Moreover, no differences were detected between the *Drosophila* components, RICN and Mam, or the murine components, NICD and MamL (Figure 3B–C’). This result is quite remarkable, indicating that the activator complex forms largely with the same efficacy regardless of whether or not RBPJ or Su(H) is present. Moreover, these data strongly emphasize that the Su(H)^LLL^/RBPJ^LLL^ variants fold correctly, thereby leaving interaction with the Notch activator complex intact. However, the reduced binding affinity of RBPJ to Hairless may be causative for the reduced repressor ability of RBPJ in the fly, resulting in the observed gain of Notch activity. 

### 3.5. Notch Activity Is Increased RBPJ^LLL^ Homozygous Mutants

**Figure 4:**
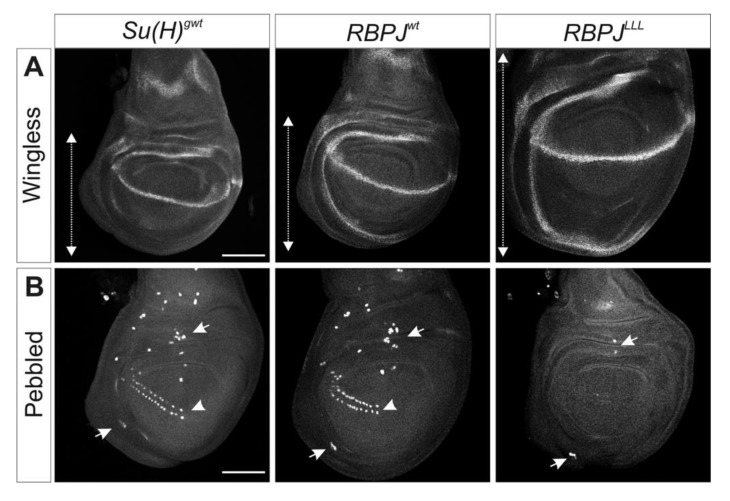
*RBPJ^LLL^* homozygotes display strong gain of Notch activity. Wing imaginal discs derived from homozygous larvae, as indicated, were assayed for (**A**) Wingless or (**B**) Pebbled (also named Hindsight) protein expression. (**A**) Compared to the control *Su(H)^gwt^*, the wing blade area is slightly enlarged in the *RBPJ^wt^* homozygote and strongly hypertrophied in the *RBPJ^LLL^* mutant discs (double headed arrow). (**B**) Sensory organ precursors express Pebbled (arrows point to examples). Their number is strongly reduced in *RBPJ^LLL^* mutant discs. Note complete absence of the presumptive triple row in the presumptive wing field, marked by an arrowhead in the controls. Size bar: 100 µm.

Loss of *Su(H)* activity results in late larval/early pupal death, at which the mutant larvae are characterized by small wing imaginal discs due to a failure to establish a robust expression of *wingless* (*wg*) along the dorso–ventral boundary [50,51]. Therefore, wing imaginal discs are well suited to assess Notch signaling activity in vivo mediated by Su(H) and RBPJ, respectively. Whereas wing imaginal discs of homozygous *RBPJ^wt^* mutant larvae were similar to wild type, those of *RBPJ^LLL^* mutant larvae were considerably larger (Figure 4A). Tissue overgrowth is a typical consequence of Notch hyperactivation [52,53], and was likewise observed in the *Su(H)^LLL^* mutants [24]. In addition, we investigated the process of lateral inhibition, i.e., the singling-out of sensory organ precursor cells in wing imaginal discs. To this end, we used Pebbled protein as a marker (also named Hindsight), a Zn-finger type transcriptional regulator that specifically accumulates in cells of neuronal fate in third instar larval discs [54,55,56]. The number of Pebbled-positive sensory organ precursors was strongly reduced in *RBPJ^LLL^* mutant wing discs, whereas those in *RBPJ^wt^* larvae were similar to the control (Figure 4B). Sensory organ precursor formation is restricted by Notch-mediated lateral inhibition, i.e., their disappearance conforms to increased Notch activity [40]. Apparently, also in the *RBPJ* homozygotes, Notch activity is increased, confirming the adult phenotypes.

**Figure 5:**
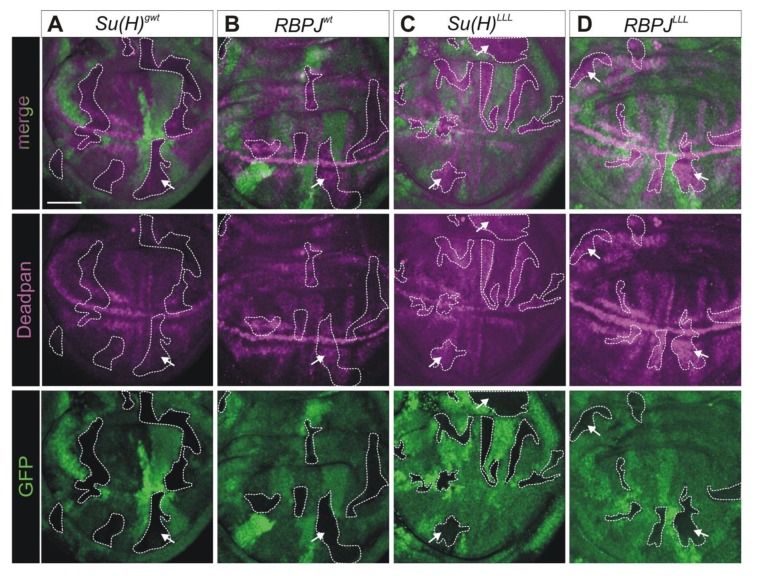
The Notch target Dpn is de-repressed in *RBPJ^LLL^* homozygous cells. (**A**–**D**) Clonal analysis to monitor expression of Deadpan (Dpn; magenta); wild type cells are labeled with GFP (green), whereas homozygous cells of the indicated genotype are unlabeled. Mutant cell clones are outlined for clarity. Dpn expression is undisturbed in *Su(H)^gwt^* control clones (**A**), but appears mildly upregulated in *RBPJ^wt^* clones (arrow) (**B**). In contrast, cell clones homozygous mutant for *Su(H)^LLL^* (**C**) or *RBP^LLL^* (**D**) display a robust upregulation of Dpn expression (arrows). Size bars: 50 µm.

In order to more directly address the gain of Notch activity in *RBPJ^wt^* and *RBPJ^LLL^* mutant cells, we analyzed the expression of the Notch regulated gene *deadpan* (*dpn*) [57,58]. Within larval wing imaginal discs, expression of *dpn* is a direct, widespread response to Notch activation. Dpn protein accumulates predominantly along the dorso–ventral boundary and in presumptive intervein regions (Figure 5) [57,59]. The Dpn protein belongs to the Hairy and Enhancer of split (HES) gene family of bHLH-O transcription factors implementing Notch responses [57,60,61].To this end, we generated cell clones homozygous for either *RBPJ^wt^* or *RBPJ^LLL^*, neighboring wild type cell clones in a heterozygous background by Flp/FRT mediated recombination (Figure 5) [62]. We expected a de-repression of *dpn* expression in case of reduced repressor complex formation by *RBPJ^wt^* or *RBPJ^LLL^*. In fact, *dpn* expression was mildly de-repressed in homozygous *RBPJ^wt^* cells (Figure 5B), an effect that was considerably stronger in either *RBPJ^LLL^* or *Su(H)^LLL^* homozygous cells (Figure 5C,D).

**Figure 6:**
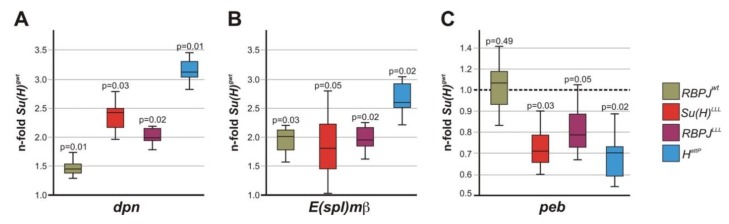
Quantification of altered transcription resulting from the failure of repressor complex formation. Expression of *dpn* (**A**), *E(spl)mß* (**B**), and *peb* (**C**) transcripts, respectively, was quantified by qRT-PCR relative to *Su(H)^gwt^*; *cyp33* and *Tbp* were used as reference genes. mRNA was prepared from larval wing discs isolated from 25 homozygous larvae, each of the indicated genotype. Data were gained from four biological and two technical replicates. An increase in *dpn* and *E(spl)mß* transcription levels was observed in *Su(H)^LLL^*, *RBPJ^LLL^,* and *H^attp^* mutants. In contrast, *peb* transcripts were reduced. Median corresponds to expression ratio; mini-max depicts 95% confidence. The p-values are given above each bar; significance was tested using PFRR from REST (*p* < 0.05).

Next, we employed qRT-PCR to determine changes of Notch dependent target gene expression in *RBPJ^wt^* and *RBPJ^LLL^* flies. We included *Su(H)^LLL^*, as well as *H^attP^* mutants, for comparison, and used *Su(H)^gwt^* as a control. mRNA was isolated from wing imaginal discs of homozygous, staged third instar larvae, and the expression profile of two well-established Notch target genes, *dpn* and *E(spl)mß* was measured: a robust increase was consistently observed in *RBPJ^wt^* compared to *Su(H)^gwt^* flies, an effect which was significantly higher in either mutant *RBPJ^LLL^*, *Su(H)^LLL^*, or *H^attP^* (Figure 6A,B). Moreover, we assayed the expression of *pebbled (peb),* which is expressed in the sensory organ precursors [55,56]. In accord with a reduced number of sensory organ precursor cells in wing discs of *RBPJ^LLL^* (Figure 4B), as well as of *Su(H)^LLL^* or *H^attP^* mutants [24], *peb* expression was reduced (Figure 6C). Note that we consistently observed the strongest deregulation of Notch targets in the *H^attP^* mutant. Together, these data demonstrate that the *RBPJ^LLL^* allele mimics the *Su(H)^LLL^* allele: Both are defective in the repression of Notch signaling activity, resulting in a strong gain of Notch function. Hence, as predicted by the structural similarities, H-repressor complex assembly takes place with either Su(H) or RBPJ. Apparently, the CTD of RBPJ can incorporate the H interaction domain just like Su(H), predicting a likewise conformational distortion of RBPJ that precludes binding of NICD [21]. Hence, respective leucine mutations within RBPJ precluding H binding, hamper repressor complex formation and result in a deregulation of Notch target genes. Most likely, the RBPJ-H repressor complex is structurally very similar to the *Drosophila* Su(H)-H repressor complex, confirming the usefulness of our model system.

### 3.6. Stability of RBPJ Protein Depends on Its Recruitment into Repressor or Activator Complexes

**Figure 7:**
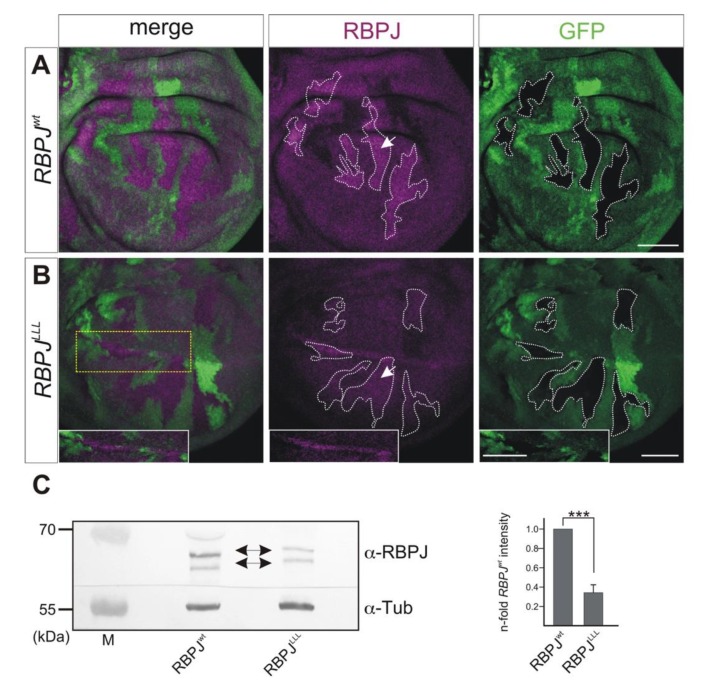
RBPJ^LLL^ protein abundance is lowered compared to RBPJ^wt^. (**A,B**) Clonal analysis: cells with a wild type *Su(H)* gene copy are labeled by GFP (green); RBPJ protein expression is shown (magenta). Homozygous RBPJ* cell clones are outlined for clarity. RBPJ protein accumulates to a higher level in homozygous *RBPJ^wt^* cells (arrow) than in heterozygous cells (*RBPJ^wt^*/+). (**B**) RBPJ protein is barely visible in the *RBPJ^LLL^* heterozygous cells, and is likewise lowered in homozygous cells (outlined). Note, however, that nuclear accumulation of RBPJ protein in a stripe of cells along the dorso–ventral boundary. Insets show enlargements of framed region. Size bars represent 50 µm. (**C**) Western blots on protein extracts from homozygous larvae: Note reduced level of RBPJ protein (double headed arrows) in the *RBPJ^LLL^* mutants compared to *RBPJ^wt^* control. M, pre-stained protein marker (in kDa). Tubulin was used as a loading control (Tub). Blot was cut for parallel detection of RBPJ and Tubulin. Quantification of signals from four independent Western blots with *Image J* gel analysis program in relation to the beta-Tubulin signals. Error bars denote standard deviation; Student’s *t* test was applied (*** *p* < 0.001). Uncropped blots used for quantification are shown in supplemental Figure S4.

As an abrogation of binding to H is correlated with reduced stability of Su(H) protein, we asked whether *RBPJ^LLL^* mutant cells also suffer from a reduced level of RBPJ protein. To this end, we generated cell clones homozygous for the *RBPJ^LLL^* mutant form and compared the level of RBPJ protein with that in the heterozygous neighboring cells bearing one copy of the wild type *Su(H)* allele. As a control, we likewise generated *RBPJ^wt^* homozygous cell clones for comparison. Intriguingly, we observed a lowered abundance of RBPJ^LLL^ mutant protein compared to the RBPJ wild type version (Figure 7A,B). These results are similar to what was observed in the *Su(H)^LLL^* mutant cells regarding Su(H) protein levels [24]. Quantification of RBPJ levels by Western blot analysis corroborated the in situ data: Compared to RBPJ wild type protein, less than 40% RBPJ^LLL^ protein was detected in extracts derived from respective homozygous larvae (Figure 7C and Figure S4). These data indicate that, in *Drosophila*, RBPJ protein is stabilized through the binding to H within the repressor complex.

In *Drosophila*, Su(H) is likewise stabilized in the activator complex by the binding to NICD. For example, Su(H)^LLL^ protein is detected along the dorso–ventral boundary in wing imaginal discs at places of highest Notch activity, or where NICD is overexpressed [24]. Unfortunately, homozygous *RBPJ^LLL^* larvae overexpressing NICD died before crawling in the third instar larval stage, precluding further analyses. RBPJ^LLL^ mutant protein, however, accumulated along the dorso–ventral boundary in wing imaginal discs (Figure 7B), suggesting that RBPJ stability may likewise depend on its binding to NICD.

## 4. Discussion

### 4.1. The RBPJ-H Repressor Complex

In this work, we have established a fly model containing the mouse *RBPJ* instead of the endogenous *Su(H)* gene. The extremely high conservation of the two proteins at the level of primary and secondary structure prompted our experiment. We demonstrated that mouse *RBPJ* can largely substitute for *Su(H)*, allowing the development of adult flies, indicating that (i) regulation of RBPJ and (ii) regulation by RBPJ matches the orthologue Su(H). This covers two completely different aspects: (i) regulation of Su(H) acts, for example, at the level of stability, i.e., availability of the protein [24,63], whereas (ii) regulation by Su(H) requires formation of multi-protein complexes and an activator, as well as repressor complexes, moreover involving chromatin regulators (reviewed e.g., in [2,5,12]). Our yeast interaction assays demonstrated no differences in activator complex formation but demonstrated reduced ability to assemble repressor complexes. Yet, despite the reduced binding affinity, the structure of the RBPJ-H repressor complex is predicted to equal the structure of Su(H)-H repressor complex, since *RBPJ^LLL^* mutation abolished H binding in the yeast and repressor activity in vivo just like the *Su(H)^LLL^* mutation [21,24]. Apparently, H can wedge itself in the immunoglobulin domain of the CTD of RBPJ similarly to that of Su(H), thereby enforcing a conformational change precluding Notch binding [21]. Thus, the peculiar and novel interaction mode of H and Su(H) described for the first time in the Notch repressor complex [21] forms likewise between H and RBPJ. Presumably, the binding takes place in mammalian cells on RBPJ protein, as well [20], establishing H as a potent candidate for therapeutic intervention of overshooting Notch signaling activity also there.

### 4.2. Regulation of Mouse RBPJ Availability in Drosophila

The principles of Notch signal transduction require CSL protein to be available at any time of Notch receptor activation [2,3,64]. Accordingly, *Su(H)* is ubiquitously expressed throughout development in all tissues analyzed [64,65,66,67]. However, manifold genetic and molecular analyses have indicated that the availability of Su(H) is restricted, despite its apparent ubiquitous presence [6,47,53,65,68]. Stability of Su(H) protein may underlie this apparent discrepancy. We already know, that Su(H) stability depends on the formation of transcription–regulator complexes, either activator complexes together with NICD or repressor complexes together with H [24]. Accordingly, Su(H) protein level is reduced in the absence of H, and likewise in the presence of a Su(H)-binding deficient H^LD^ variant. Moreover, the H-binding deficient Su(H)^LLL^ protein is barely detected except at places of high Notch activity [24]. Additionally, Su(H) protein availability in the nuclear compartment depends on its cofactors NICD and H [63,69,70,71].

RBPJ appears to follow the same regulatory rules in *Drosophila* tissue, since RBPJ^LLL^ protein has impaired stability like its Su(H)^LLL^ orthologue. We conclude, that RBPJ is protected from degradation by its partners within transcription–regulator complexes. If RBPJ underlies the same regulatory mechanisms like Su(H), it must be likewise targeted by specific proteases or the proteasome, perhaps upon specific secondary modification/s. If secondary modification/s are involved, the relevant enzymes must equally recognize RBPJ or Su(H) in the *Drosophila* tissue. Earlier, it was demonstrated that RBPJ is an unstable protein with a half-life of roughly two hours. Degradation has been linked to phosphorylation at position threonine 378 by MAPK p38, modulated by Presenilin 2 [72]. In *Drosophila*, the corresponding residue threonine 426 in Su(H) is also targeted by MAPK, and phosphorylation at this site impedes Notch signaling activity [73]. However, stability of phosphorylated Su(H) appeared unaffected. Rather, the secondary modification influenced the dynamics of repressor or activator complex formation or its transition, providing a means of crosstalk between the Notch- and the EGFR-signaling pathway [73]. The relevance of T378 phosphorylation on RBPJ turnover can now be addressed in vivo in the fly system by introducing specific mutations.

### 4.3. Transcriptional Regulation of Notch Target Genes by RBPJ in Drosophila

*RBPJ^wt^* homozygotes display Notch gain of function phenotypes affecting the development of mechanosensory bristle organs, the wings, and the male genitalia. Some of these defects, notably in wing venation and bristle development, are characteristic of the haplo-insufficient *H* phenotype [26,43,45]. They are in accordance with the reduced binding affinity of RBPJ to H. Similar phenotypes are also seen in the antimorphic *N^Ax^* alleles that display increased Notch signaling activity [4,43,74]. Rotated male genitalia, however, are not a typical outcome of a general increase in Notch signaling activity. This phenotype results from the overexpression or accumulation of NICD specifically in genital discs [42], suggesting a more context-specific defect in *RBPJ^wt^* flies. Perhaps the recruitment of certain co-factors by RBPJ protein differs from Su(H). These may be tissue-specific co-regulators or, alternatively, context-specific chromatin modifiers. Specific transcription factors cooperating with Su(H) and Notch may elicit activity differences and eventually determine cell lineage decision in a context-specific manner (reviewed in [2,75]). For example, Notch cooperates with Runx transcription factors in blood cell lineages both in mammals and in *Drosophila* [76,77] (reviewed in [2,75]). It remains to be determined whether these transcription factors are interchangeable and whether gene activation responses are similar. Su(H) engages with other highly conserved transcription factors also in other contexts, but without known vertebrate parallels. For example, the differentiation of a bristle socket cell (the base of a fly’s external mechanoreceptor) relies on the cooperative activity of Su(H) and Ventral veins lacking (vvl), a POU-homeodomain transcription factor of Pou-III family [78]. Perhaps Su(H) engages with another well-conserved transcription factor in cells of the genital disc. In case this conserved transcription factor is recruited by RBPJ, albeit with a stronger affinity, transcriptional activation of Notch target genes would be increased. Such a scenario may explain a higher Notch activity, specifically in this organ, resulting in mis-rotation of the genitalia [42]. As Notch target gene responses are strongly influenced by the chromatin environment, it is also conceivable that changes in chromatin accessibility affect the morphogenesis of genital discs in *RBPJ^wt^* males. In mammals, the chromatin landscape of Notch target genes is shaped by RBPJ-associated factors, activators, and repressors, which may be regulated by post-translational modifications themselves, thereby integrating signaling inputs from other pathways [7] (reviewed in [2,5,12]). Hence, tissue-specific differences in chromatin regulation may explain altered developmental outcomes as seen for the genital disc. This hypothesis could also explain the slightly different responses of the Notch target genes to the deregulation in *RBPJ^wt^* cells that we recorded by qPCR. In this case, our RBPJ^wt^ fly model may serve to uncover such tissue-specific factors, their roles in Su(H) mediated Notch gene regulation, and the role of their mammalian homologues. 

### 4.4. Perspectives of the RBPJ Fly Model

*Drosophila* has served, and serves as, a model for a variety of human pathologies (for review e.g., [79,80]). Moreover, the *Drosophila* model system was extremely useful in unraveling the Notch signaling pathway (for review e.g., [2,81]). We have generated a partly ‘mammalian-like’ fly model to study RBPJ function in vivo in the context of development, in whole tissue and united cell structures, allowing further directed manipulations. Albeit, the *RBPJ^wt^* flies are not completely like the wild type, as murine RBPJ allows completion of all developmental stages to adulthood, granting further analyses. Moreover, our model allows RBPJ to be changed along known mutations linked to disease, addressing, for example, (suspected) biochemical properties in vivo. These may include secondary modifications, like phosphorylation, as outlined above, acetylation, and ubiquitylation. Moreover, other Notch pathway components might be exchanged with mouse homologues in the longer run, to follow Notch signaling activity and regulation in vivo in the fly.

## 5. Conclusions

*Drosophila* is well suited as a model to study the function and regulation of mammalian components of the Notch signaling pathway in the in vivo developmental context. We showed that *Drosophila* Su(H) can be replaced by murine RBPJ, allowing for a largely normal development of adult flies, despite the fact that RBPJ shows a reduced binding to the fly-specific repressor H. In fact, activity of RBPJ depends on H recruitment as an H-binding defective RBPJ^LLL^ variant is incompatible with normal fly development. Moreover, we demonstrated that the stability of RBPJ protein depends on the assembly in either activator or repressor complexes, suggesting a likewise regulation of RBPJ availability in mammals, as in flies. Overall, our work opens a new avenue in the in vivo study of murine RBPJ in a large variety of tissues and developmental contexts.

## Supplementary Materials

The following supplementary figures are available online at https://www.mdpi.com/2073-4409/8/10/1252/s1: Figure S1. Sequence comparison between fly Su(H) and murine RBPJ protein; Figure S2. Substitution of murine *RBPJ* for *Su(H)* in the fly by genome engineering; Figure S3. Defective adult genitalia rotation in *RBPJ^wt^* males; and Figure S4. Uncropped blots used for RBPJ protein quantification.
