# Supplementary material for: An RBPJ-Drosophila Model Reveals Dependence of RBPJ Protein Stability on the Formation of Transcription–Regulator Complexes
Source: Cells. 2019 Oct 14;8(10):1252. doi: 10.3390/cells8101252 (PMC6829621; doi:10.3390/cells8101252)
Supplement: Supplementary file 1 [file cells-08-01252-s001.pdf]

Supplemental Figures to

An RBPJ-*Drosophila* model reveals dependence of RBPJ protein stability on the formation of transcription-regulator complexes

Bernd M. Gahr [1,2], Franziska Brändle [1], Mirjam Zimmermann [1], Anja C. Nagel [1,*]

[1]Institute of Genetics (240), University of Hohenheim, Garbenstr. 30, 70599 Stuttgart, Germany

[2]Present address: Molecular Cardiology, Department of Internal Medicine II, University of Ulm, Albert-Einstein-Allee 23, 89081 Ulm, Germany

[*]: Correspondence: anja.nagel@uni-hohenheim.de

The supplemental Figures contain

Figure S1: Sequence comparison between fly Su(H) and murine RBPJ protein,

Figure S2: Substitution of murine *RBPJ* for *Su(H)* in the fly by genome engineering,

Figure S3: Defective adult genitalia rotation in $RBPJ^{wt}$ males,

Figure S4: Uncropped blots used for RBPJ protein quantification.

Figure S1 Sequence comparison between fly Su(H) and murine RBPJ protein

```
Dm   1 MKSYSQFNLNAAAPPAIAYETTVVNPNGSPLDPHQQQQQQSQDMPHFGLP      50
                               .|:|.|..|....:.:    |...:.
Mm   1 ------------------------MPSGFPQSPRTSPRAR----PKTRIT      22

Dm  51 GPQPPSSQQQQQQLQVHHQQQQQQQQQQQQQQHQQQMQMSLLPGPYRPHI     100
       |..|              ....:....::...|...      .|.:....
Mm  23 GALP--------------MDYSEGLSAEERPAHAPS------AGKFGERP      52

Dm 101 EEKKLTRDAMEKYMRERNDMVIVILHAKVAQKSYGNEKRFFCPPPCIYLF     150
       ..|:|||:||..|::||.|..::|||||||||||||||||||||||:||.
Mm  53 PPKRLTREAMRNYLKERGDQTVLILHAKVAQKSYGNEKRFFCPPPCVYLM     102

Dm 151 GSGWRRRYEEMLQQGEGEQGAQLCAFIGIGSSDQDMQQLDLNGKQYCAAK     200
       ||||:::.|:|.:.|..||.:|.||||||||:|||:||||:|.||.||.||
Mm 103 GSGWKKKKEQMERDGCSEQESQPCAFIGIGNSDQEMQQLNLEGKNYCTAK     152

Dm 201 TLFISDSDKRKHFMLSVKMFYGNGHDIGVFNSKRIKVISKPSKKKQSLKN     250
       ||:||||||||||||||||||||..|||||.|||||||||||||||||||
Mm 153 TLYISDSDKRKHFMLSVKMFYGNSDDIGVFLSKRIKVISKPSKKKQSLKN     202

Dm 251 ADLCIASGTNVALFNRLRSQTVSTRYLHVENGHFHASSTQWGAFTIHLLD     300
       |||||||||.||||||||||||||||||||.|:|||||.|||||.|||||
Mm 203 ADLCIASGTKVALFNRLRSQTVSTRYLHVEGGNFHASSQQWGAFYIHLLD     252

Dm 301 DNESESEEFQVRDGYIHYGATVKLVCSVTGMALPRLIIRKVDKQMALLEA     350
       |:|||.|||.|||||||||.||||||||||||||||||||||||.|||:|
Mm 253 DDESEGEEFTVRDGYIHYGQTVKLVCSVTGMALPRLIIRKVDKQTALLDA     302

Dm 351 DDPVSQLHKCAFYMKDTDRMYLCLSQEKIIQFQATPCPKEPNKEMINDGA     400
       |||||||||||||:|||:|||||||||:|||||||||||||.||||||||
Mm 303 DDPVSQLHKCAFYLKDTERMYLCLSQERIIQFQATPCPKEQNKEMINDGA     352

Dm 401 CWTIISTDKAEYQFYEGMGPVASPVTPVPIVNSLNLNGGGDVAMLELSGD     450
       .|||||||||||.||||||||.:||||||:|.||.||||||||||||:|.
Mm 353 SWTIISTDKAEYTFYEGMGPVLAPVTPVPVVESLQLNGGGDVAMLELTGQ     402

Dm 451 NFTPHLQVWFGDVEAETMYRCTETLLCVVPEISQFRGEWLWVRQPTQVPI     500
       ||||:|:||||||||||||||.|::|||||:||.||..|.|||||.|||:
Mm 403 NFTPNLRVWFGDVEAETMYRCGESMLCVVPDISAFREGWRWVRQPVQVPV     452

Dm 501 SLVRNDGIIYATGLTFTYTPEPGPRPHCNTQAEDVMRARQN---------     541
       :||||||:||:|.|||||||||||||||:. |..::||..:
Mm 453 TLVRNDGVIYSTSLTFTYTPEPGPRPHCSA-AGAILRANSSQVPSNESNT     501

Dm 542 NNNNNITSISNNNNSNNAGSPAAGGGLQQQQQQHQALPSISEVQWNSHGSGLS 594
       |:..|.|:.|.|:.|..:.:.....
Mm 502 NSEGNYTNASTNSTSVTSSTATVVS                             526
```

A bestfit comparison of protein sequence, performed with the *EMBOSS* needle program: *D. melanogaster* Su(H) [upper row], *M. musculus* RBPJ [lower row]. Overall identity is 61%, and similarity 69.8%. In bold and underlined are the amino acids fused in the fly construct. Note high conservation within the N-terminal domain (light blue), the β-trefoil domain (green) and the C-terminal domain (yellow). The N-terminally located α1-helix (yellow) makes contact to the C-terminal

domain (see Fig. 1A), which is connected to the β-trefoil domain by a β strand (grey). Red color highlights the three leucine residues at position 434, 445 and 514 in Su(H) known to contact H in the repressor complex; their replacement by alanine prevents Su(H)-H repressor complex formation. The respective leucines in RBPJ at position 386, 397 and 466 are labeled in blue.

Figure S2 Substitution of murine *RBPJ* for *Su(H)* in the fly by genome engineering

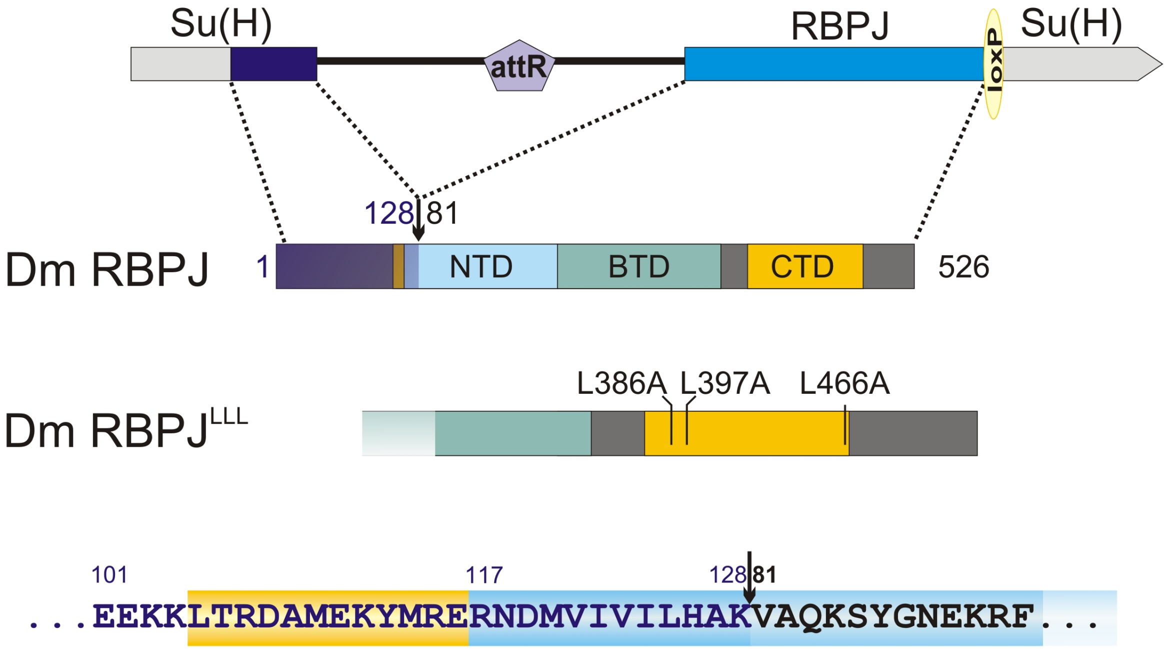

Subsequent to genome engineering, a gene fusion between Su(H) and RBPJ results in the formation of a fusion protein containing RBPJ from valine 81 fused to the N-terminal 128 amino acids of Su(H). Hence α1-helix and the start of NTD are derived from Su(H), which is extremely similar to RBPJ in this part (58% identity and 79% similarity; see Figure S1 for a sequence comparison). The sequence of the fusion is depicted, color-coded as in Figure S1. $RBPJ^{LLL}$ contains three leucine mutations at position 386, 397 and 466, which were replaced by alanine.

Figure S3: Defective adult genitalia rotation in *RBPJ*^{wt} males

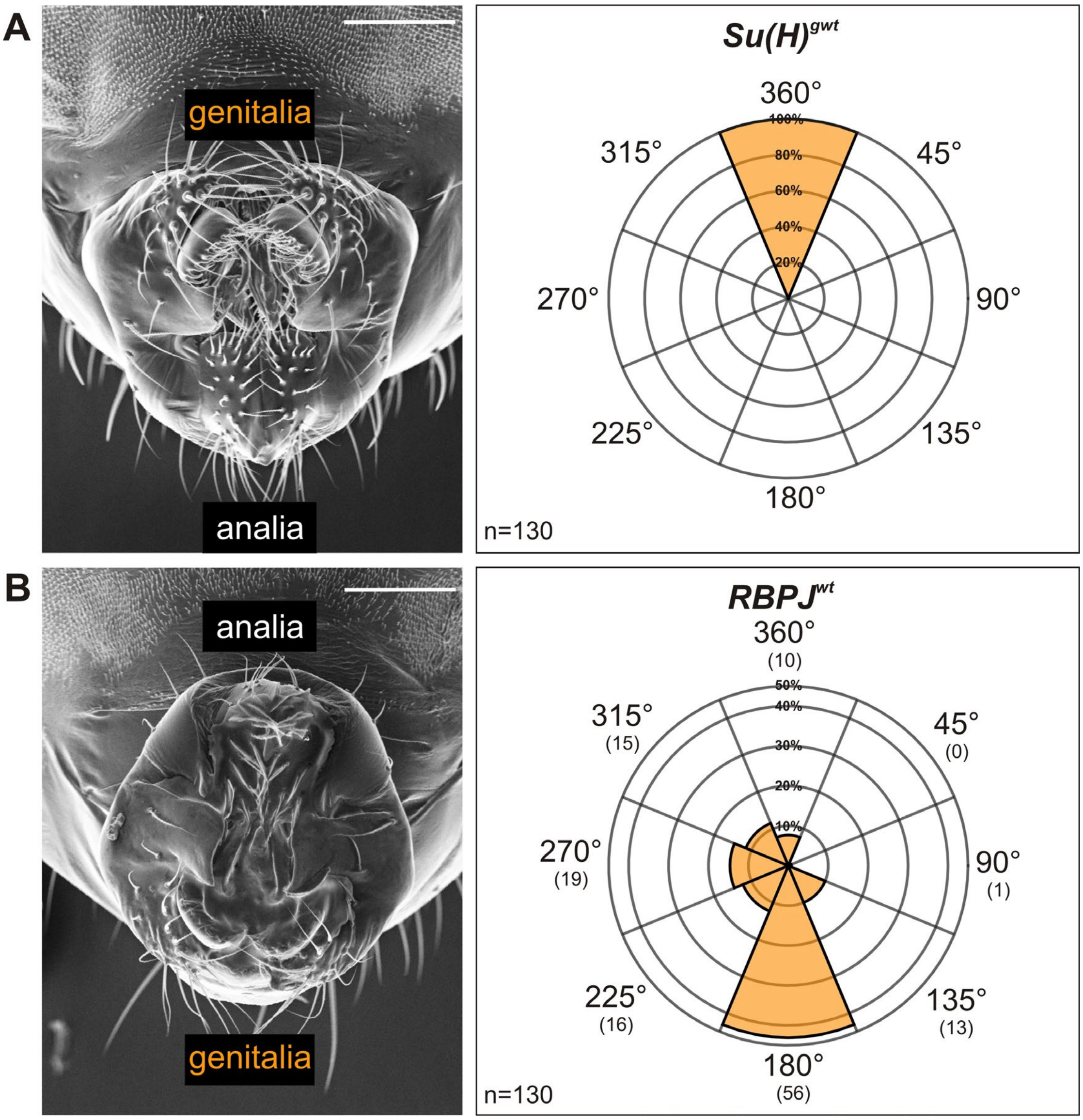

(**A**) *Su(H)*^{gwt} adult male genitalia. (**B**) Rotated genitalia are observed in *RBPJ*^{wt} homozygous males with high frequency. Scanning electron micrographs show enlargements of the terminal abdomen with the genitalia and analia from *Su(H)*^{gwt} control (A) and a half rotated *RBPJ*^{wt} (B). Most frequently, rotation stopped half way at an 180° angle, as shown in the chart (polarhistograph) (n=130). Scale bars: 100 µm.

Figure S4 Uncropped blots used for RBPJ protein quantification

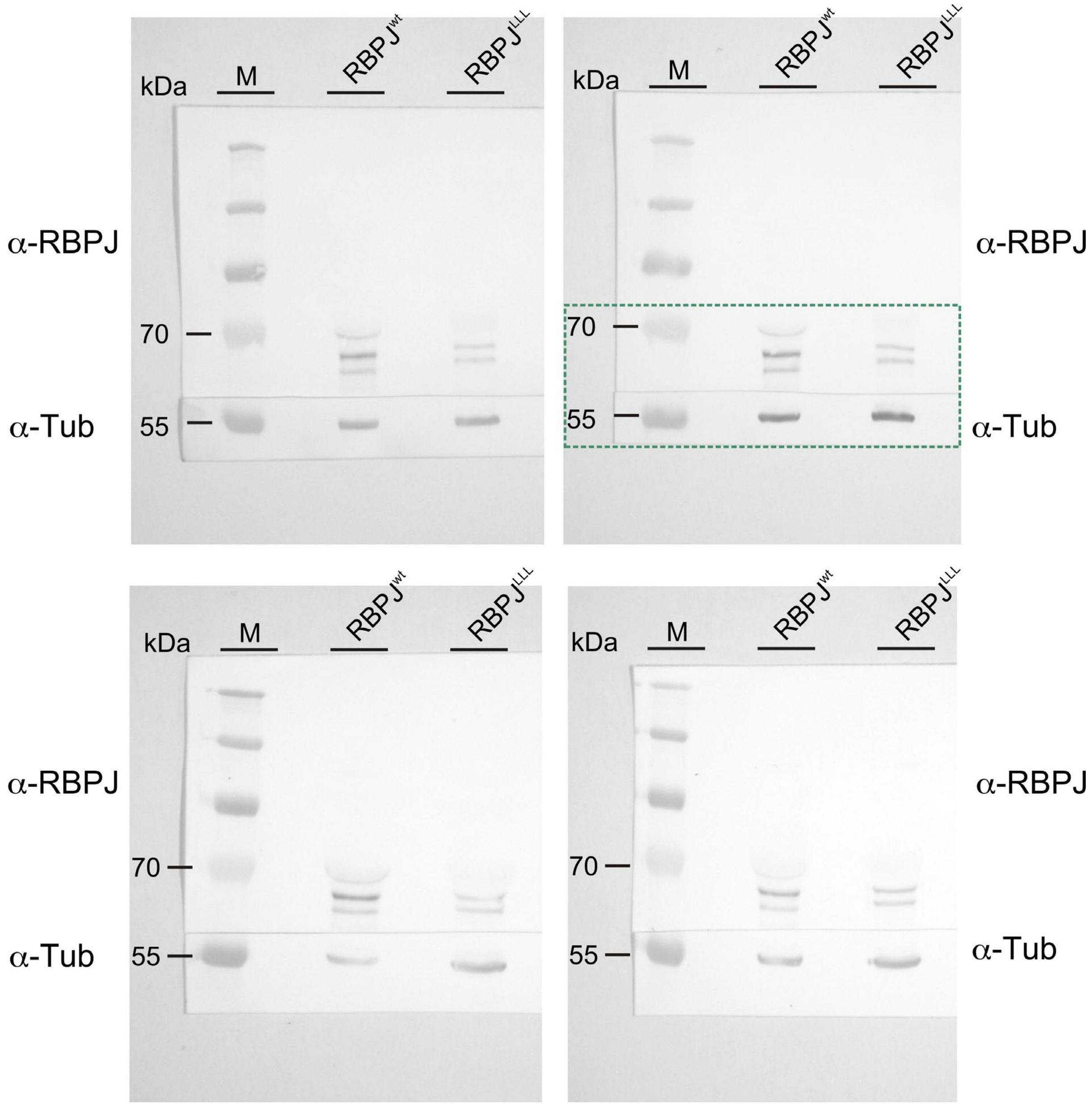

Original blots used for quantification of RBPJ levels in Figure 7C. The blot depicted in Figure 7C is framed. Blots were cut for parallel detection of RBPJ and Tubulin; the latter served as internal standard.
